# Granular Inflammation of the Eyes

**Published:** 1874-07

**Authors:** 


### GRANULAR INFLAMMATION OF THE EYES.

A PROMINENT oculist says that the contagious Egyptian or granular inflammation of the eyes is spreading rapidly throughout the country, and adds: "I have in many, and I may say in the majority of the cases, been able to trace the disease to the use of the so-called rolling towels. Such towels are generally found in our country hotels, and sleeping apartments of the working classes, and being thus used by nearly every one, are made the carrier of one of the most dangerous, and, as regards its symptoms, most troublesome diseases of the eye. I, therefore, would strongly recommend that the use of the rolling towel be abolished, for thereby we will discard one of the great instruments for the spread of such a dangerous disease of the eye, by which thousands of workingmen are annually deprived of their means of support."

